# Regional Odontodysplasia: A Systematic Review of Case Reports

**DOI:** 10.3390/ijerph19031683

**Published:** 2022-02-01

**Authors:** Kacper Nijakowski, Patryk Woś, Anna Surdacka

**Affiliations:** 1 Department of Conservative Dentistry and Endodontics, Poznan University of Medical Sciences, 60-812 Poznan, Poland; annasurd@ump.edu.pl; 2 Student’s Scientific Group, Department of Conservative Dentistry and Endodontics, Poznan University of Medical Sciences, 60-812 Poznan, Poland; patrykwos4@gmail.com

**Keywords:** regional odontodysplasia, dental anomaly, ghost teeth, systematic review, case reports

## Abstract

Regional odontodysplasia is a rare developmental disorder characterised by hypoplasia and hypomineralisation of enamel and dentin. Our systematic review aimed to organise the knowledge on localisation, symptomatology and treatment methods in patients with regional odontodysplasia based on case reports published in the databases PubMed, Scopus and Web of Science. Case reports were described in 28 different countries, considering 180 patients (including 91 females). Regional odontodysplasia occurs mainly in both deciduous and permanent dentition (66.1%). The affected teeth were observed more frequently in the maxilla (70.0%), especially on the left side (45.6%). The most common reported symptoms were ghost teeth, poorly developed buds, yellowish-brown colour of crowns and delayed eruption of permanent teeth in affected quadrants. The most popular treatment method was surgical treatment (78.6%) with subsequent prosthetic therapy (34.6%). Based on the review of cases, pathognomonic clinical and radiological signs can be found, however, it is difficult to reach a consensus on the choice of treatment method.

## 1. Introduction

Regional odontodysplasia (RO) is a rare developmental anomaly characterised by hypoplasia and hypocalcification of dental hard tissues. The condition was probably first described by Hitchin in 1934, but the term was first used in 1963 by Zegarelli et al. [1]. Among three case reports, most of the anomalous teeth were unerupted and intraosseously located, as well as the erupted teeth were also poorly developed. It was observed that the calcification of enamel and dentin had appeared to be severely deficient in both quality and quantity. The prefix “regional” was added by Pindborg in 1970, considering its localised nature [2,3]. It usually affects one quadrant; however, cases only affecting one tooth or generalised have been reported in the literature [4].

The altered teeth are commonly yellow or brown and show abnormal morphology. Most patients suffer from recurrent purulent inflammations or tooth mobility. Wide pulp chambers, open apices and blurred demarcation in the dentino-enamel junction are the main radiological features distinctive of RO [5]. The disorder involves both primary and permanent dentitions in the majority of the cases. Histologically, both enamel and dentin appear hypomineralised with poorly organised dentinal tubules and enamel prisms. The pulp chambers very often contain calcifications in the coronary area [6].

The aetiology remains still unknown. Several factors have been suggested in the literature, such as teratogenic medications used during pregnancy, latent viruses, circulatory disorders, radiation, hyperpyrexia accompanying severe childhood disease, nutritional deficiencies, Rh disease, local trauma or ischemia [7]. Both trauma and circulatory disorders seem to be relevant, as they have appeared several times as possible factors among the case reports [8,9,10,11]. There are some examples of hereditary prevalence suggesting genetic causes in some of the patients. Koskinen et al. [12] described a 6.5-year-old girl, whose mother and two sisters (all of the siblings) had partially missing teeth with no other symptoms. On the contrary, there are also cases highlighting the absence of a positive familial history in the affected patients [13,14]. Even though many authors suggest the potential cause, no general conclusions have been made.

**Figure 1:**
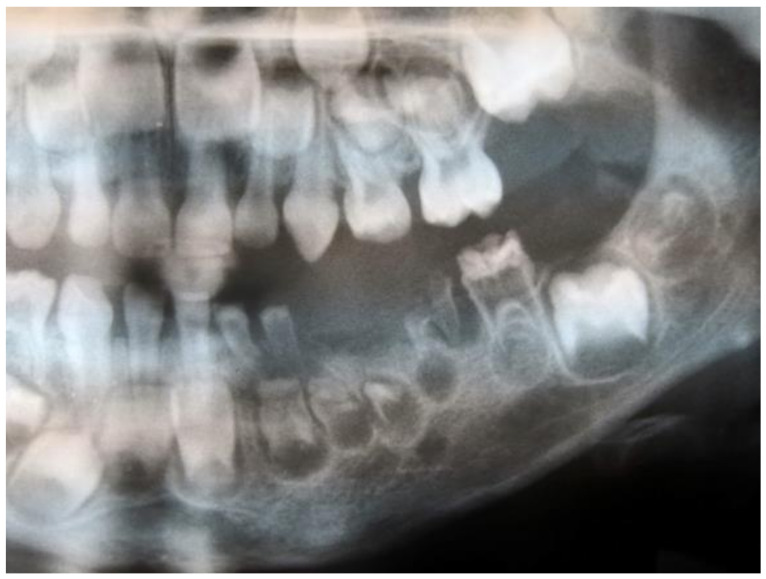
Fragment of the panoramic radiograph showing “ghost teeth” (in the mandibular left quadrant) characteristic of regional odontodysplasia—personal collection (K.N., Poland, 2019).

There are relatively few case reports of regional odontodysplasia in the literature, e.g., in Poland we are the first to describe this anomaly [15]. A 5-year-old male patient was referred to our department. The main complaint was recurrent purulent inflammations of deciduous teeth in the third quadrant. The affected teeth differed from the healthy because of their yellowish-brown discolouration and tendency to crumble rapidly. The pregnancy, medical and family histories were unremarkable. Other teeth did not present any pathology. Based on the panoramic radiograph taken three months before the appointment, the mandibular left deciduous teeth and the follicles of succedaneous permanent teeth demonstrated a “ghost-like” appearance (Figure 1). Moreover, the underdevelopment of the left side of the mandible and the slight shift of the midline to the left were noticeable. The periapical radiographs presented blurred demarcation between thin layers of enamel and dentin in the affected teeth. Additionally, wide pulp chambers with open apices were visible for “ghost teeth”. The proposed treatment was the surgical removal of the residual roots of the primary mandibular left first molar. A week later, on a follow-up visit, the active fistula and increased mobility were observed in the primary left incisors. Therefore, they were extracted too. After a month, it was decided to remove the last deciduous tooth in this quadrant because of severe pain during biting. Due to the early loss of affected primary teeth, the patient was referred for a removable acrylic partial denture. Two years after the first visit, the follow-up panoramic radiograph presented slight progress in the development of the permanent teeth in the affected region.

Our systematic review aimed to organise the existing knowledge on localisation, symptomatology and treatment methods in patients with regional odontodysplasia based on published case reports.

## 2. Materials and Methods

This systematic review was conducted up to 1 September 2021, according to the Preferred Reporting Items for Systematic Reviews and Meta-Analyses (PRISMA) statement guidelines [16], using the databases PubMed, Scopus and Web of Science. The search formula included “odontodysplasia” or “regional odontodysplasia” or “ghost teeth” as MeSH (medical subject headings) terms combined in PubMed Advanced Search Builder. In other databases, the same keywords were used (in Scopus as index terms and in Web of Science as author keywords or keywords plus).

**Figure 2:**
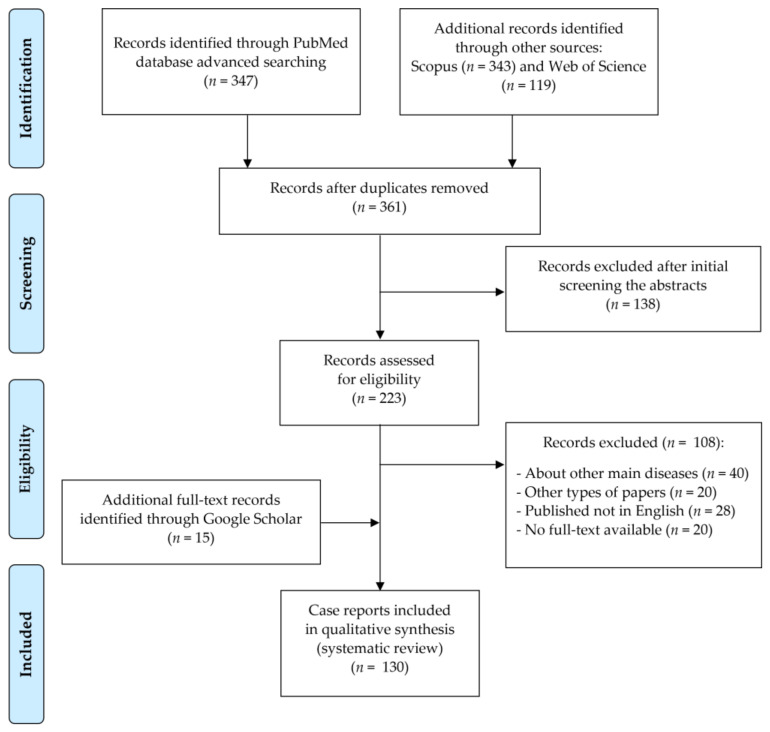
PRISMA flow diagram presenting the detailed search strategy.

Records were screened by the title, abstract and full text by two independent investigators. Studies included in this review matched all the predefined criteria according to the PICOS (“Population”, “Intervention”, “Comparison”, “Outcomes”, “Study design”)—Table 1. A detailed search flowchart is presented in Figure 2 (in Section 3).

The critical appraisal of case reports was assessed according to the JBI Critical Appraisal Tool issued by the Faculty of Health and Medical Sciences at the University of Adelaide, South Australia. These questionnaires were answered by two independent investigators, and disagreements were resolved by discussion between them. Due to the rarity of the condition, the decision was made to include all papers dealing with regional odontodysplasia and containing basic patient information, including necessarily the location of the condition. Among the included papers, one report was without the gender of the patient, 21 patients without a detailed macroscopic image of the affected teeth, 14 patients without an attached radiograph, and 21 patients without the treatment method used (with only diagnostic description of the patients). Additionally, additional histopathologic findings were presented in 80 patients.

All included studies have a 4th level of evidence, according to the classification of the Oxford Center for Evidence-Based Medicine levels for diagnosis [17].

## 3. Results

In this systematic review, 130 papers following the search criteria were included (the list is attached as Supplementary Materials). Case reports were described in 28 different countries, considering a total of 180 patients (including 91 females). Among them, 74 contained long-term descriptions of clinical observations. Figure 2 shows the detailed selection strategy of the articles. The inclusion and exclusion criteria are presented in Table 1 (in Section 2).

Regardless of the sex of the patients, regional odontodysplasia occurs in both deciduous and permanent dentition (66.1%). The affected teeth were observed more frequently in the maxilla (70.0%), especially on the left side (45.6%). Generalised odontodysplasia was described in only 10 patients (5.5%). The distribution of locations in the included cases is presented in Table 2.

In the clinical examination, the most common findings were yellowish-brown colour of crowns (90.6%) and delayed eruption of permanent teeth (90.6%). Moreover, the radiographs practically always showed ghost teeth (100.0%) and poorly developed buds (92.2%) in affected quadrants. The presence of other features is shown in Table 3. Taking into account gender, in female patients swelling was observed significantly more often, as well as local pain and mobility differed closely to statistical significance. Radiologically, no differences in the frequency of symptoms in patients of different sexes were noted.

Based on Table 4, the most popular treatment method used in patients with odontodysplasia was surgical treatment (78.6%) accompanied by prosthetic treatment (34.6%). Similarly, according to gender, no significant differences were found in the distributions of individual treatment methods.

Table 5 shows the histopathological changes found in the involved teeth. Irregular calcification of interglobular dentin (92.5%) and hypoplasia or hypomineralisation of enamel (77.5%) were the most frequently observed.

## 4. Discussion

Regional odontodysplasia is a condition that we still know very little about. There are only suggestions of possible aetiology, and therefore there is no possibility of preventing its emergence. The purpose of this review was to collect data from all available case reports, including those reported in the recent past and draw conclusions based on the results compared to similar works from the past.

As seen in the table, the condition appears more often in the maxilla (61.1%) than in the mandible (30%); the remaining cases had symptoms both in the upper and lower jaws (8.9%). The predilection is similar to what was found by previous authors [18,19]. However, the collected data shows that there is no significant sex predilection of regional odontodysplasia. On the contrary, other literature gives ratios as high as 1.37:1, 1.4:1 and even 1.7:1 of female to male cases, which shows a big difference from our results presented in this review (1.03:1) [18,19,20]. The possible explanation for this disparity can be a high number of new case reports taken into account in this article compared to older works. 

It is worth noting that usually regional odontodysplasia affects quadrants or a quadrant as a whole, however, there are some examples where teeth from one quadrant and just one tooth from another one crossing the midline are affected. This was the case in a report by Courson et al. from 2003. An 11-year-old boy was diagnosed with “ghost teeth” in the upper right maxilla and the left central incisor was also affected [21]. The same was observed in a 12-year-old girl described by Carlos et al. in 2008 [22]. It is difficult to draw any concrete conclusions from this observation, but it is possible that this manifestation could indicate a different etiological factor than the rest.

In general, all of the typical known clinical symptoms were observed in the collected group. The most common, which appeared in over 90% of the patients, were yellowish-brown colour and delayed teeth eruption, but they varied in the extent. It is also possible that even more patients than stated in the table had these manifestations but were not reported by the authors either because the teeth were lost before seeking medical attention or were not noted by the clinicians. The main radiological symptom was the appearance of “ghost teeth” reported in all cases and poorly developed buds for patients with unerupted teeth (92.2%). This finding concludes that radiographs should remain the main diagnostic method confirming RO. Local pain is reported by 44.7% of the patients. The majority do not experience any direct pain associated with the disorder. It would explain why, very often, especially in less developed countries, patients seek medical attention in later life when the teeth are already lost. The oldest patient of all the collected cases was 35 years old at the time of the diagnosis [23].

The histopathological examination of the extracted teeth shows that the most common feature is irregular calcification of dentin, followed by hypoplastic and hypomineralised enamel. It is worth pointing out that some of the examined teeth lacked the enamel layer, lost due to grinding or caries [24]. Not many, only 8.8%, of the reports included dislocated cementum present on the surface of the crown. It is difficult to say whether this feature is more common for the disorder but was not reported for the same reasons as stated above or is simply rare among the affected teeth.

In our case of regional odontodysplasia, spectrometric analysis was conducted on the extracted molar. The results show a significantly lower magnesium to calcium ratio in the dysplastic tooth compared to the control healthy tooth in the exfoliation period. The same can be said about sodium to calcium ratio (data not published).

Most of the patients had characteristics of regional odontodysplasia in both dentitions, but in 28.9% only the secondary dentition was affected and in 5% only the primary. This tendency suggests that the etiological factor is present for a longer time in the majority of the cases, assuming the factor can influence the teeth in the germ formation and development period. There have been reports of only one tooth being affected and, on the other hand, generalised cases [9,25]. This prevalence excludes the possibility of the cause being purely systemic and implies possible etiological factors that result in a similar condition manifestation. In one of the cases an 8.5-year-old boy was diagnosed with generalised odontodysplasia [26]. All primary and secondary teeth had visible changes on the radiograph indicative of ghost teeth. The patient also suffered from other genetic disorders—dolichocephaly and clinodactyly. There was no history of trauma or other events that could be suspected of being the main cause of either of the conditions. This suggests there is a possibility of RO being determined genetically at least in some of the cases. Another example of generalised RO is a 5-year-old girl that, on the other hand, had a very significant medical history [27]. At 2.5 months old, she was diagnosed with irregular breathing problems and was operated on for a right diaphragm hernia. Two months later she was hospitalised due to an RS-virus infection. In this case, both primary and secondary dentitions were affected, the same as in the previously described one.

An important etiological factor that was mentioned several times in the literature is local trauma. In 2021, Majumdar et al. [28] described a case of a 10-year-old girl with RO of front secondary teeth in the mandible. The patient suffered a trauma to the affected region at the age of 2 months. A similar case was described in 2014 by Ramakrishnan and Menon [9]. A girl, 7 years old at the time of the diagnosis, had characteristics of regional odontodysplasia only in one tooth—the maxillary right central incisor. The girl had a history of intrusive trauma of the corresponding primary tooth, which was extracted shortly after the incident. There is one described patient, where trauma during pregnancy could have influenced the development of the condition. In a case presented by Volpato et al. [8] in 2008, a 12 year-old-female with history of being born with a partially swollen and reddish face along with a deviation of the chin to the right. The mother fell during the third trimester of pregnancy. Otherwise, she was completely healthy and there was no facial asymmetry, the only supposed consequence of the incident was “ghost teeth” in the mandibular region, crossing the midline.

The treatment of RO varies depending on the severity of the disorder. Some patients can be successfully treated by conservative restorations or root canal treatment [29], but the most common method is the extraction of the affected teeth performed in 78.6% of the patients. It is worth noting that in some cases, other possible treatment methods were applied, and in the event of no success, the extraction turned out to be necessary [26]. There are examples of patients who were treated with implantoprosthetics (7.5%). Therefore preserving the teeth is important to promote the development of the bone to make it possible when they reach adulthood [30,31]. Regional odontodysplasia very often causes malocclusion, shift of the midline to the affected side and orthodontic treatment is then necessary [14,32]. As patients get older, their aesthetic expectations play a bigger role. In many cases, a partial acrylic denture is made not only to restore the correct occlusion but also to improve the appearance. 

Another interesting solution is autotransplantation of tooth germs into the area, where extractions were performed, and was used in several patients with success [33,34,35]. The oldest case described in 1993 involved a 10-year-old girl [33]. She was referred to remove unerupted maxillary left incisors and canine due to their malformation manifested by little demarcation between enamel and dentin, as well as wide pulp chambers and root canals with open apices. After autogenous bone graft, mandibular second premolars were autotransplanted in the affected frontal area. The longest documented case of regional odontodysplasia treated by autotransplantation had a 6-year follow-up [34]. This report presented a 9-year-old girl who suffered from recurrent periapical inflammations in the mandibular right quadrant. Clinically, the causal teeth were hypoplastic with soft yellowish enamel, as well as radiographically, these teeth were manifested as “ghost teeth”. The radical treatment with surgical removal of almost all teeth in the affected area under general anaesthesia was conducted. Only fully erupted incisors with RO were not extracted. Immediately, the first premolars from the three other quadrants were autotransplanted into the right side of the mandible. Annual postoperative radiographs showed normal condition of periodontal ligaments and alveolar bone. At the follow-up visit after 6 years, no percussion abnormalities and radiographic signs of ankylosis or external resorption of the autotransplanted teeth were observed. Thanks to the successful surgery, the symmetrical development of the mandible had been preserved. The last case with performed autotransplantation from 2012 demonstrated a boy with RO confined to the anterior mandibular region on the left side [35]. This report described the combined surgical and orthodontic treatment. In the 2-step autotransplantation, the second maxillary left premolar was transplanted in place of the removed canine and then, after a year and a half, the second maxillary right premolar was implanted in place of the removed incisors. After concomitant orthodontic treatment, a good aesthetic as well as functional effect was achieved.

Undoubtedly, the strength of our systematic review is the analysis of all case reports available in English regarding regional odontodysplasia. However, it is difficult to draw unambiguous conclusions, because the papers concern a rare disorder that is often not correctly diagnosed in everyday clinical work. Case reports are classified as having a low-evidence level and currently it is difficult to publish papers of this kind in reputable journals, which affects their reduced availability. Moreover, the case reports were published in a wide time period and have a high heterogeneity. Not all of them contained the attached full clinical picture and radiological examination, and some of them described only the diagnostic issues without performing any therapeutic methods. Additionally, the authors of the papers were not always characterised by academic affiliation, which could affect the completeness of the presented data. Despite the aforementioned limitations resulting from the individual nature of the included case reports, the above review collected and summarised the knowledge published so far about regional odontodysplasia.

## 5. Conclusions

Regional odontodysplasia is a rare developmental anomaly affecting deciduous and permanent teeth, which for several decades has been described only by authors from a few countries. Nevertheless, the radiological picture of ghost teeth accompanying clinical discolouration of tooth crowns due to enamel hypoplasia can be considered as a pathognomonic symptom. Although there is no consensus on the treatment method of choice, most cases ended with the surgical removal of the affected teeth. Similarly, the potential etiopathogenesis of this disorder has not been determined so far. It must be emphasised that patients with regional odontodysplasia require long-term specialised care due to the rapid onset and progression of the disease, as well as the gradual need to restore aesthetic and functional properties of the dentition.

## Figures and Tables

**Table 1:** Inclusion and exclusion criteria according to the PICOS.

Parameter	Inclusion Criteria	Exclusion Criteria
Population	patients with regional odontodysplasia—aged from 0 to 99 years, both sexes	patients with other developmental dental anomalies
Intervention	not applicable	
Comparison	not applicable	
Outcomes	clinical description with radiological features and/or histopathological analyses	only histopathological or another analysis without clinical description
Study design	case reports	literature reviews, expert opinion, letters to editor, conference reports, case–control, cohort and cross-sectional studies
	published up to September 2021	not published in English

**Table 2:** Location of teeth affected by regional odontodysplasia (%).

		All *n* = 180	Male *n* = 88	Female *n* = 91	*p*-Value
dentition	primary	5.0	3.4	6.6	0.479
	secondary	28.9	29.5	27.5	
	both	66.1	67.1	65.9	
jaw	maxilla	61.1	58.0	63.7	0.680
	mandible	30.0	34.0	26.4	
	both	8.9	8.0	9.9	
quadrants	1st	44.4	47.7	40.7	0.281
	2nd	45.6	37.5	53.8	0.048 *
	3rd	25.6	27.3	24.2	0.665
	4th	28.9	26.1	31.9	0.497
affected region	left side of maxilla	25.0	18.2	31.9	0.362
	right side of maxilla	21.7	23.8	18.7	
	left side of mandible	10.0	15.9	4.4	
	right side of mandible	11.7	12.5	11.0	
	bilateral maxilla	14.4	15.9	13.2	
	bilateral mandible	8.3	5.7	11.0	
	ipsilateral left quadrants	0.0	0.0	0.0	
	ipsilateral right quadrants	1.7	2.3	1.1	
	contralateral quadrants	0.0	0.0	0.0	
	without one quadrant	1.7	2.3	1.1	
	generalised	5.5	3.4	7.6	

* *p*-value <0.05 for the Chi-square test.

**Table 3:** Clinical and radiological features in patients with regional odontodysplasia (%).

**Clinical Features**	**All** ***n* = 159**	**Male** ***n* = 76**	**Female** ***n* = 83**	***p*-Value**
yellowish-brown colour	90.6	89.5	91.6	0.652
gingival swelling	67.3	55.3	78.3	0.002 *
underdeveloped one side of maxilla/mandible	17.6	21.1	14.5	0.276
active fistulas	18.9	18.4	19.3	0.890
tooth mobility	41.5	34.2	48.2	0.074
early loss of primary teeth	47.8	50.0	45.8	0.595
delayed tooth eruption	90.6	93.4	88.0	0.239
local pain	44.7	36.8	51.8	0.058
**Radiological features**	**All** ***n*** **= 166**	**Male** ***n*** **= 79**	**Female** ***n*** **= 87**	***p*** **-Value**
ghost teeth	100	100	100	-
poorly developed buds	92.2	91.1	93.1	0.638
periapical lesions	39.2	40.5	37.9	0.734

* *p*-value <0.05 for the Chi-square test.

**Table 4:** Treatment methods in patients with regional odontodysplasia (%).

Treatment Method	All *n* = 159	Male *n* = 75	Female *n* = 83	*p*-Value
conservative restoration	14.5	18.7	10.8	0.164
steel crown	5.7	8.0	3.6	0.235
pulpotomy/RCT	2.5	4.0	1.2	0.264
tooth extraction	78.6	74.7	83.1	0.191
acrylic partial denture	34.6	40.0	30.1	0.193
orthodontic treatment	16.4	21.3	12.0	0.116
implantoprosthetic treatment	7.5	6.7	8.4	0.676
autotransplantation	1.9	1.3	2.4	0.621

**Table 5:** Histopathological features in patients with regional odontodysplasia (%).

Histopathological Features	*n* = 80
hypoplastic and hypomineralised enamel	77.5
irregular calcification of dentin	92.5
irregular dentino–enamel junction	28.8
pulpal calcifications	45.0
dislocated cementum	8.8

## Data Availability

Data are available on request from the corresponding author.

## Supplementary Materials

The following are available online at https://www.mdpi.com/article/10.3390/ijerph19031683/s1, the list of included case reports (in alphabetic order).
