# Supplementary material for: Regional Odontodysplasia: A Systematic Review of Case Reports
Source: Int J Environ Res Public Health. 2022 Feb 1;19(3):1683. doi: 10.3390/ijerph19031683 (PMC8835375; doi:10.3390/ijerph19031683)
Supplement: Supplementary file 1 [file ijerph-19-01683-s001.zip › ijerph-1529663-supplementary.pdf]

Supplementary material – the list of included case reports (in alphabetic order)

1. Abdel-Kader MA, Abdelazeem AF, Ahmed NE-MB, Khalil YM, Mostafa MI. Oral rehabilitation of a case with regional odontodysplasia using a regenerative approach-A case report and a review of literature. Spec Care Dentist 2019;39:330–9. doi:10.1111/scd.12378.

2. Abrams AM, Groper J. Odontodysplasia. Report of three cases. J Dent Child 1966;33:353–62.

3. Al-Mullahi AM, Toumba KJ. Regional Odontodysplasia with Generalised Enamel Defect. Case Rep Dent 2016;2016:4574673. doi:10.1155/2016/4574673.

4. Al-Tuwirqi A, Lambie D, Seow WK. Regional odontodysplasia: literature review and report of an unusual case located in the mandible. Pediatr Dent 2014;36:62–7.

5. Alexander WN, Lilly GE, Irby WB. Odontodysplasia. Oral Surg Oral Med Oral Pathol 1966;22:814–20. doi:10.1016/0030-4220(66)90372-0.

6. Anneroth G, Ramström G. Unilateral odontodysplasia. Swed Dent J 1980;4:93–100.

7. Ansari G, Reid JS, Fung DE, Creanor SL. Regional odontodysplasia: report of four cases. Int J Paediatr Dent 1997;7:107–13. doi:10.1111/j.1365-263x.1997.tb00288.x.

8. Babar P, Baig BU, Maxood A, Bashir U. Regional odontodysplasia: report of an unusual case. Pakistan Orthodontic Journal 2015;7:41–4.

9. Babu NSV, Smriti RJ, Pratima DB. Regional odontodysplasia: Report of an unusual case involving mandibular arch. Contemp Clin Dent 2015;6:237–9. doi:10.4103/0976-237X.156054.

10. Badger GR. Regional Odontodysplasia - An Unusual Development. DOHC 2016;1:1–4. doi:10.24966/DOHC-6783/100001.

11. Bagherpoor MR, Siadat H, Nokar S, Alikhasi M. Step-by-step oral rehabilitation of a generalized odontodysplastic patient with implant-supported prostheses: a clinical report. Implant Dent 2010;19:122–7. doi:10.1097/ID.0b013e3181d43b16.

12. Barbería E, Sanz Coarasa A, Hernández A, Cardoso-Silva C. Regional odontodysplasia. A literature review and three case reports. Eur J Paediatr Dent 2012;13:161–6.

13. Bowden E, Carroll C, Gill M, Llewelyn R. Cervicofacial infection in a 3-year-old child with regional odontodysplasia: A case report and literature review. J Oral Maxillofac Pathol 2018;22:S117–20. doi:10.4103/jomfp.JOMFP_146_17.

14. Burch MS, Besley KW, Samuels HS. Regional odontodysplasia with associated midline mandibular cyst: report of case. J Oral Surg 1973;31:44–8.

15. Cabral LA, Carvalho YR, Moraes E, Nogueira T de O, Cavalcante AS, de Moraes LC. Regional odontodysplasia: a report of three cases. Quintessence Int 1994;25:141–5.

16. Cahuana A, González Y, Palma C. Clinical management of regional odontodysplasia. Pediatr Dent 2005;27:34–9.

17. Canela AHC, Rezende KMPEC, Benitez M, Bönecker M. Early diagnosis of regional odontodysplasia in an infant. J Craniofac Surg 2012;23:e134-136. doi:10.1097/SCS.0b013e31824cdb15.

18. Carlos R, Contreras-Vidaurre E, Almeida OP de, Silva KR, Abrahão PG, Miranda AMMA, et al. Regional odontodysplasia: morphological, ultrastructural, and immunohistochemical features of the affected teeth, connective tissue, and odontogenic remnants. J Dent Child (Chic) 2008;75:144–50.

19. Chaudhry AP, Wittich HC, Stickel FR, Holland MR. Odontogenesis imperfecta. Report of a case. Oral Surg Oral Med Oral Pathol 1961;14:1099–103. doi:10.1016/0030-4220(61)90503-5.

20. Chinn C, Kohli K. Regional odontodysplasia. A case report. N Y State Dent J 2003;69:27–9.

21. Cho S. Conservative management of regional odontodysplasia: case report. J Can Dent Assoc 2006;72:735–8.

22. Clark SL, Cairns AM, Hunter KD. Comparative case report of segmental odontomaxillary dysplasia and regional odontodysplasia. Dental Update 2014;41:825–31. doi:10.12968/denu.2014.41.9.825.

23. Courson F, Bdeoui F, Danan M, Degrange M, Gogly B. Regional odontodysplasia: expression of matrix metalloproteinases and their natural inhibitors. Oral Surg Oral Med Oral Pathol Oral Radiol Endod 2003;95:60–6. doi:10.1067/moe.2003.59.

24. Dagistan S, Miloglu O, Goregen M, Harorli A. Regional Odontodysplasia: A Case Report. JODDD 2009;3:141–4.

25. Dahllöf G, Lindskog S, Theorell K, Ussisoo R. Concomitant regional odontodysplasia and hydrocephalus. Oral Surg Oral Med Oral Pathol 1987;63:354–7. doi:10.1016/0030-4220(87)90204-0.

26. Damasceno JX, Couto JLP, Alves KS da S, Chaves CM, Costa FWG, Pimenta A de MV, et al. Generalized odontodysplasia in a 5-year-old patient with Hallermann-Streiff syndrome: clinical aspects, cone beam computed tomography findings, and conservative clinical approach. Oral Surg Oral Med Oral Pathol Oral Radiol 2014;118:e58-64. doi:10.1016/j.oooo.2014.04.013.

27. de Sá Cavalcante D, Fonteles CS, Ribeiro TR, Kurita LM, Pimenta AVM, Sr Carvalho F, et al. Mandibular Regional Odontodysplasia in an 8-year-old Boy showing Teeth Disorders, Gubernaculum Tracts, and Altered Bone Fractal Pattern. Int J Clin Pediatr Dent 2018;11:128–34. doi:10.5005/jp-journals-10005-1498.

28. Fanibunda KB, Soames JV. Odontodysplasia, gingival manifestations, and accompanying abnormalities. Oral Surg Oral Med Oral Pathol Oral Radiol Endod 1996;81:84–8. doi:10.1016/s1079-2104(96)80154-5.

29. Ferguson FS, Creath CJ, Buono B. Infraorbital infection related to odontodysplasia: case report. Pediatr Dent 1990;12:397–400.

30. Ferguson F, Schlissel E, Kucine A, Alexander S, DeSantis A, Hendricks R, et al. Long-term management of a child with regional odontodysplasia: a case report. Pediatr Dent 2009;31:346–9.

31. Ferguson JW, Geary CP. Regional odontodysplasia. Aust Dent J 1980;25:148–51. doi:10.1111/j.1834-7819.1980.tb03705.x.

32. Fujiwara T, Nakano K, Sobue S, Ooshima T. Simultaneous occurrence of unusual odontodysplasia and oligodontia in the permanent dentition: report of a case. Int J Paediatr Dent 2000;10:341–7. doi:10.1046/j.1365-263x.2000.00217.x.

33. Galeone RJ, Philips JF, Pincock DG. Odontodysplasia. Oral Surg Oral Med Oral Pathol 1970;29:879–80. doi:10.1016/0030-4220(70)90442-1.

34. Ganguly R, Ramesh A. Regional odontodysplasia: a case of progressive tooth development. J Indian Soc Pedod Prev Dent 2012;30:176–8. doi:10.4103/0970-4388.100010.

35. Gardner DG, Sapp JP. Regional odontodysplasia. Oral Surg Oral Med Oral Pathol 1973;35:351–65. doi:10.1016/0030-4220(73)90073-x.

36. Gerlach RF, Jorge J, de Almeida OP, Coletta RD, Zaia AA. Regional odontodysplasia. Report of two cases. Oral Surg Oral Med Oral Pathol Oral Radiol Endod 1998;85:308–13. doi:10.1016/s1079-2104(98)90014-2.

37. Gibbard PD, Lee KW, Winter GB. Odontodysplasia. Br Dent J 1973;135:525–32. doi:10.1038/sj.bdj.4803111.

38. Gomes MP, Modesto A, Cardoso AS, Hespanhol W. Regional odontodysplasia: report of a case involving two separate affected areas. ASDC J Dent Child 1999;66:203–7, 155.

39. Gondim JO, Pretel H, Ramalho LTO, Santos-Pinto LAM, Giro EMA. Regional odontodysplasia in early childhood: a clinical and histological study. J Indian Soc Pedod Prev Dent 2009;27:175–8. doi:10.4103/0970-4388.57099.

40. Goodridge DL. GHOST TEETH OR ODONTO DYSPLASIA. J R Nav Med Serv 1964;50:162–7.

41. Gümrü OZ, Kasaboğlu C, Koçak H, Canbaz AE, Alatli C, Olgaç V. Regional odontodysplasia. J Nihon Univ Sch Dent 1993;35:209–12. doi:10.2334/josnusd1959.35.209.

42. Gündüz K, Zengin Z, Celenk P, Ozden B, Kurt M, Gunhan O. Regional odontodysplasia of the deciduous and permanent teeth associated with eruption disorders: A case report. Med Oral Patol Oral Cir Bucal 2008;13:E563-566.

43. Gurunathan D, Tandon S, Krishnan RR, Kalra M. Solitary odontodysplasia: a rare entity. J Indian Soc Pedod Prev Dent 2011;29:S56-60. doi:10.4103/0970-4388.90743.

44. Guzman R, Elliott MA, Rossie KM. Odontodysplasia in a pediatric patient: literature review and case report. Pediatr Dent 1990;12:45–8.

45. Hainline-Raez AG, Richardson DS. Abnormal odontogenesis: report of case. ASDC J Dent Child 1985;52:130–3.

46. Hamdan MA, Sawair FA, Rajab LD, Hamdan AM, Al-Omari IKH. Regional odontodysplasia: a review of the literature and report of a case. Int J Paediatr Dent 2004;14:363–70. doi:10.1111/j.1365-263X.2004.00548.x.

47. Hanks PA, Williams B. Odontodysplasia: report of two cases. Pediatr Dent 1998;20:199–203.

48. Hegazi F, Hassan M. Regional Odontodysplasia Crossing the Midline. J Dent Child (Chic) 2018;85:88–91.

49. Hess P, Lauridsen EF, Daugaard-Jensen J, Worsaae N, Kofod T, Hermann NV. Treatment Strategies for Patients with Regional Odontodysplasia: A Presentation of Seven New Cases and a Review of the Literature. Oral Health Prev Dent 2020;18:669–81. doi:10.3290/j.ohpd.a45070.

50. Hintz CS, Peters RA. Odontodysplasia. Report of an unusual case and a review of the literature. Oral Surg Oral Med Oral Pathol 1972;34:744–50. doi:10.1016/0030-4220(72)90292-7.

51. Hovinga J, Ingenhoes R. Regional odontodysplasia. Int J Oral Surg 1979;8:474–7. doi:10.1016/s0300-9785(79)80088-5.

52. Ibrahim Mostafa M, Samir Taha N, Ismail Mehrez MA. Generalised versus Regional Odontodysplasia: Diagnosis, Transitional Management, and Long-Term Followup—A Report of 2 Cases. Case Reports in Dentistry 2013;2013:1–5. doi:10.1155/2013/519704.

53. Ide M, Oshima Y, Chiba T, Adaniya A, Kuroki T, Asada Y. Regional odontodysplasia in maxillary right anterior region–First report: Clinical management in relation to 2 case reports. Pediatric Dental Journal 2020;30:124–8. doi:10.1016/j.pdj.2020.01.002.

54. Iizawa F, Kinjoh N, Taguchi Y. Regional odontodysplasia: Long-term observation of a case on the mandibular left side. pediatric dental journal 2010;20:103–9. doi:10.11411/pdj.20.103.

55. Jahanimoghadam F, Pishbin L, Rad M. Clinical, Radiographic, and Histologic Evaluation of Regional Odontodysplasia: a Case Report with 5-year Follow-up. J Dent (Shiraz) 2016;17:159–63.

56. Jeffery S, Brock GR, Harrison JE, Butterworth CJ. Multidisciplinary Management of Maxillary Regional Odontodysplasia. Orthodontic Update 2019;12:8–12. doi:10.12968/ortu.2019.12.1.8.

57. Jensen JL, Ambjornsen E, Haanaes HR, Storhaug K. Case report: Unclassified syndrome involving dental enamel, dentine and lack of tooth eruption. Eur Arch Paediatr Dent 2009;10:244–7. doi:10.1007/BF03262691.

58. Jindal M, Faridi A, Hashmi G, Ahmad M. Odontodysplasia- A case report. Indian Dental Research and Review 2008;57–8.

59. Kahn MA, Hinson RL. Regional odontodysplasia. Case report with etiologic and treatment considerations. Oral Surg Oral Med Oral Pathol 1991;72:462–7. doi:10.1016/0030-4220(91)90561-p.

60. Kannan SK, Saraswathi K. Regional odontodysplasia (Ghost teeth). A case report. Indian J Dent Res 2001;12:242–6.

61. Kappadi D, Ramasetty PA, Rai KK, Rahim AMB. Regional odontodysplasia: An unusual case report. J Oral Maxillofac Pathol 2009;13:62–6. doi:10.4103/0973-029X.57671.

62. Kinirons MJ, O'Brien FV, Gregg TA. Regional odontodysplasia: an evaluation of three cases based on clinical, microradiographic and histopathological findings. Br Dent J 1988;165:136–9. doi:10.1038/sj.bdj.4806520.

63. Koruyucu M, Yaman D, Seymen F, Demirel K, Gençay K. Management of regional odontodysplasia: a 10-year-follow-up case report and literature review. Eur Oral Res 2018;52:111–6. doi:10.26650/eor.2018.495.

64. Koskinen S, Keski-Filppula R, Alapulli H, Nieminen P, Anttonen V. Familial oligodontia and regional odontodysplasia associated with a PAX9 initiation codon mutation. Clin Oral Investig 2019;23:4107–11. doi:10.1007/s00784-019-02849-5.

65. Kuklani RM, Supancic JS, Cohen DM, Bhattacharyya I. Improperly formed maxillary teeth. J Am Dent Assoc 2010;141:1346–50. doi:10.14219/jada.archive.2010.0079.

66. Leonard M, McClure I. Odontodysplasia. A case report. J Dent 1972;1:43–5. doi:10.1016/0300-5712(72)90042-5.

67. Lian CB, Chong BS, Siar CH, Phang YC. Ghost teeth. Case report. Aust Dent J 1988;33:291–4. doi:10.1111/j.1834-7819.1988.tb04179.x.

68. Lima SAA, de Oliveira AV, Lima LSG, Paranhos LR, Jóias RP, Siqueira DF. Characteristics of regional odontodysplasia: A case report. Acta Scientiarum - Health Sciences 2012;34:215–9. doi:10.4025/actascihealthsci.v34i2.13380.

69. Lowry L, Welbury RR, Soames JV. An unusual case of regional odontodysplasia. Int J Paediatr Dent 1992;2:171–6. doi:10.1111/j.1365-263x.1992.tb00031.x.

70. Lustmann J, Klein H, Ulmansky M. Odontodysplasia. Report of two cases and review of the literature. Oral Surg Oral Med Oral Pathol 1975;39:781–93. doi:10.1016/0030-4220(75)90039-0.

71. Magalhães AC, Pessan JP, Cunha RF, Delbem ACB. Regional odontodysplasia: case report. J Appl Oral Sci 2007;15:465–9. doi:10.1590/s1678-77572007000600002.

72. Majumdar S, Uppala D, Priyanka K, Kumar S. Bilateral Regional Odontodysplasia: A Rare Case Report. Oral & Maxillofacial Pathology Journal 7:755–7.

73. Malhotra R, Shashikiran N, Singla S, Verma A. Regional odontodysplasia: A classical case report. SRM J Res Dent Sci 2013;4:86–9. doi:10.4103/0976-433X.120186.

74. Marques AC, Castro WH, do Carmo MA. Regional odontodysplasia: an unusual case with a conservative approach. Br Dent J 1999;186:522–4. doi:10.1038/sj.bdj.4800157.

75. Mathew A, Dauravu LM, Reddy SN, Kumar KR, Venkataramana V. Ghost teeth: Regional odontodysplasia of maxillary first molar associated with eruption disorders in a 10-year-old girl. J Pharm Bioallied Sci 2015;7:S800-803. doi:10.4103/0975-7406.163570.

76. Matsubara Y, Matsubara M, Iinuma M, Tamura Y. Clinical Management of Regional Odontodysplasia. Dent Open J 2014;1:1–4. doi:10.17140/DOJ-1-101.

77. Matsuyama J, Tanaka R, Iizawa F, Sano T, Kinoshita-Kawano S, Hayashi-Sakai S, et al. Clinical and radiographic findings and usefulness of computed tomographic assessment in two children with regional odontodysplasia. Case Rep Dent 2014;2014:764393. doi:10.1155/2014/764393.

78. Mehta DN, Bailoor D, Patel B. Regional odontodysplasia. J Indian Soc Pedod Prev Dent 2011;29:323–6. doi:10.4103/0970-4388.86380.

79. Melamed Y, Harnik J, Becker A, Shapira J. Conservative multidisciplinary treatment approach in an unusual odontodysplasia. ASDC J Dent Child 1994;61:119–24.

80. Miller WA, Seymour RH. Odontodysplasia. Br Dent J 1968;125:56–9.

81. Miloglu O, Goregen M, Akgul HM, Harorli A. Generalized familial crown resorptions in unerupted teeth. Eur J Dent 2011;5:206–9.

82. Mukhopadhyay S, Roy P, Halder M. Regional odontodysplasia in the primary dentition associated with eruption failure. Int J Health Allied Sci 2016;5:195–7.

83. N C S, Panat S, Choudhary A, Aggarwal A, Gupta P. Regional Odontodysplasia- A Case Report. Journal of Dental Sciences and Oral Rehabilitation 2011;2:24–5.

84. Naik LRK, Shetty P, Girish Babu K. Regional odontodysplasia crossing the midline: A unique case associated with bilateral impacted supernumerary teeth. J Pediatr Dent 2014;2:96. doi:10.4103/2321-6646.145585.

85. Neupert EA, Wright JM. Regional odontodysplasia presenting as a soft tissue swelling. Oral Surg Oral Med Oral Pathol 1989;67:193–6. doi:10.1016/0030-4220(89)90329-0.

86. Nijakowski K, Surdacka A. Regional odontodysplasia – a rare developmental dental anomaly. Pediatr Pol 2021;96:153–5. doi:10.5114/polp.2021.107402.

87. O'Neil DW, Koch MG, Lowe JW. Regional odontodysplasia: report of case. ASDC J Dent Child 1990;57:459–61.

88. Oncag O, Eronat C, Sen BH. Regional odontodysplasia: a case report. J Clin Pediatr Dent 1996;21:41–6.

89. Ozer L, Cetiner S, Ersoy E. Regional odontodysplasia: report of a case. J Clin Pediatr Dent 2004;29:45–8.

90. Pandis N, Polido C, Bell WH. Regional odontodysplasia. A case associated with asymmetric maxillary and mandibular development. Oral Surg Oral Med Oral Pathol 1991;72:492–6. doi:10.1016/0030-4220(91)90566-u.

91. Parmar N, Garg S, Shah S. Conservative Approach in Management of Regional Odontodysplasia. Dentimedia Journal of Dentistry 2013;18:43–6.

92. Pinkham JR, Burkes EJ. Odontodysplasia. Oral Surg Oral Med Oral Pathol 1973;36:841–50. doi:10.1016/0030-4220(73)90336-8.

93. Ponranjini VC, Jayachandran S, Bakyalakshmi K. Regional odontodysplasia: report of a case. J Dent Child (Chic) 2012;79:26–9.

94. Prakash SMR, Gupta S, Kamarthi N, Goel S. Inflammatory linear verrucous epidermal nevus and regional odontodysplasia: A rare sorority. Indian J Dent 2015;6:203–6. doi:10.4103/0975-962X.160348.

95. Pruhs RJ, Simonsen CR, Sharma PS, Fodor B. Odontodysplasia. J Am Dent Assoc 1975;91:1057–66. doi:10.14219/jada.archive.1975.0520.

96. Quinderé LB, Cavalcante RB, Nonaka CFW, Miguel MC da C, de Souza LB. Regional odontodysplasia involving three quadrants of the jaws: a case report. Quintessence Int 2010;41:13–6.

97. Raez AG. Unilateral regional odontodysplasia with ipsilateral mandibular malformation. Oral Surg Oral Med Oral Pathol 1990;69:720–2. doi:10.1016/0030-4220(90)90355-v.

98. Ramakrishnan M, Menon P. Odontodysplasia involving single tooth: A rare entity. SRM J Res Dent Sci 2014;5:140–2.

99. Rashidian A, Afsharian Zadeh M, Azarshab M, Zarrabian T. Regional Odontodysplasia: Report of a case. J Dent (Shiraz) 2013;14:197–200.

100. Reade PC, Radden BG, Barke JJ. Regional odontodysplasia. A review and a report of two cases. Aust Dent J 1974;19:152–61. doi:10.1111/j.1834-7819.1974.tb05032.x.

101. Reever JS, King WC. Unilateral maxillary odontodysplasia. ASDC J Dent Child 1971;38:23–8.

102. Rosa MCT, Marcelino GA, Belchior RS, Souza APP, Parizotto SCOL. Regional odontodysplasia: report of case. J Clin Pediatr Dent 2006;30:333–6. doi:10.17796/jcpd.30.4.x52484224j37h4v5.

103. Rushton MA. ODONTODYSPLASIA: 'GHOST TEETH'. Br Dent J 1965;119:109–13.

104. Sadeghi EM, Ashrafi MH. Regional odontodysplasia: clinical, pathologic, and therapeutic considerations. J Am Dent Assoc 1981;102:336–9. doi:10.14219/jada.archive.1981.0042.

105. Scariot R, Morosini I, Pereira CT, Amenabar JM, Rebellato N, Gugisch R. Regional odontodysplasia: A case report. Revista Odonto Ciencia 2012;27:246–50. doi:10.1590/S1980-65232012000300014.

106. Schmid-Meier E. Unilateral odontodysplasia with ipsilateral hypoplasia of the mid-face. A case report. J Maxillofac Surg 1982;10:119–22. doi:10.1016/s0301-0503(82)80023-4.

107. Shah N, Gupta YK. Generalized odontodysplasia--a case report. J Indian Soc Pedod Prev Dent 1998;16:40–3.

108. Sibley LC, Zimmermann ER. Odontogenic dysplasia. Report of a case. Oral Surg Oral Med Oral Pathol 1962;15:1370–3. doi:10.1016/0030-4220(62)90357-2.

109. Silva Cunha JL, Barboza Santana AV, Alves da Mota Santana L, Meneses Santos D, de Souza Amorim K, Maciel de Almeida Souza L, et al. Regional Odontodysplasia Affecting the Maxilla. Head Neck Pathol 2020;14:224–9. doi:10.1007/s12105-019-01031-3.

110. Slootweg PJ, Meuwissen PR. Regional odontodysplasia in epidermal nevus syndrome. J Oral Pathol 1985;14:256–62. doi:10.1111/j.1600-0714.1985.tb00489.x.

111. Sousa AS de, Nogueira JSE, Mattos SL de, Alves Júnior S de M. Regional odontodysplasia. RGO.Revista Gaúcha de Odontologia (Online) 2012;60:247–51.

112. Spini TH, Sargenti-Neto S, Cardoso SV, Souza KCN, de Souza SOM, de Faria PR, et al. Progressive dental development in regional odontodysplasia. Oral Surg Oral Med Oral Pathol Oral Radiol Endod 2007;104:e40-45. doi:10.1016/j.tripleo.2007.02.027.

113. Srindhi G, Raghavendra SS. Regional odontodysplasia: Report of a rare case and review of literature. Journal of International Dental and Medical Research 2011;4:145–9.

114. Srivathsa SH. Regional odontodysplasia: A case with radiographic evidence of advancing development. Int J Orofac Res 2018;3:37–9. doi:10.4103/ijofr.ijofr_11_18.

115. Steiman HR, Cullen CL, Geist JR. Bilateral mandibular regional odontodysplasia with vascular nevus. Pediatr Dent 1991;13:303–6.

116. Syrek DW, Gleason GA, Witkin E. Odontodysplasia. Oral Surg Oral Med Oral Pathol 1972;34:841. doi:10.1016/0030-4220(72)90304-0.

117. Tervonen SA, Stratmann U, Mokrys K, Reichart PA. Regional odontodysplasia: a review of the literature and report of four cases. Clin Oral Investig 2004;8:45–51. doi:10.1007/s00784-003-0245-0.

118. Thimma Reddy BV, Vinay Reddy KK, Sunil B, Pujita R, Kiran K, Kranthi KR. Regional odontodysplasia. J Indian Soc Pedod Prev Dent 2010;28:315–8. doi:10.4103/0970-4388.76166.

119. Turgut MD, Çelik B, Nazikoğlu A, Ataç AS, Müftüoğlu S. Clinical, radiographical and histological evaluation of an unusual regional odontodysplasia case [Sıradışı bir rejyonel odontodisplazi vakasmm

klinik, radyografik ve histolojik olarak değerlendirilmesi]. Turkiye Klinikleri Journal of Medical Sciences 2011;31:274–9. doi:10.5336/medsci.2009-13335.

120. Uner O, Yücel-Eroğlu E, Karaca I. Delayed calcification and congenitally missing teeth. Case report. Aust Dent J 1994;39:168–71. doi:10.1111/j.1834-7819.1994.tb03087.x.

121. Upadhyay V, Chaturvedi TP, Pandey RK, Chaurasia A, Singh P. Regional Odontodysplasia Crossing Midline: A Rare Case Report. Int J Clin Pediatr Dent 2011;4:159–61. doi:10.5005/jp-journals-10005-1102.

122. van der Wal JE, Rittersma J, Baart JA, van der Waal I. Regional odontodysplasia: report of three cases. Int J Oral Maxillofac Surg 1993;22:356–8. doi:10.1016/s0901-5027(05)80667-8.

123. Volpato L, Botelho G, Casela L, Borges A, Silva K. Regional odontodysplasia: report of a case in the mandible crossing the midline. J Contemp Dent Pract 2008;9:142–8.

124. von Arx T. Autotransplantation for treatment of regional odontodysplasia. Case report with 6-year follow-up. Oral Surg Oral Med Oral Pathol Oral Radiol Endod 1998;85:304–7. doi:10.1016/s1079-2104(98)90013-0.

125. Walton JL, Witkop CJ, Walker PO. Odontodysplasia. Report of three cases with vascular nevi overlying the adjacent skin of the face. Oral Surg Oral Med Oral Pathol 1978;46:676–84. doi:10.1016/0030-4220(78)90464-4.

126. Williams SA, High AS. Odontodysplasia associated with orbital coloboma. Br Dent J 1988;164:390–4. doi:10.1038/sj.bdj.4806458.

127. Wilson PHR, Ali A. Case report: restorative options in regional odontodysplasia. Eur J Prosthodont Restor Dent 2002;10:5–8.

128. Yuan SH, Liu PR, Childers NK. An alternative restorative method for regional odontodysplasia: case report. Pediatr Dent 1997;19:421–4.

129. Zegarelli EV, Kutscher AH, Applebaum E, Archard HO. Odontodysplasia. Oral Surg Oral Med Oral Pathol 1963;16:187–93. doi:10.1016/0030-4220(63)90031-8.

130. Ziegler S, Neukam FW. Regional odontodysplasia: orthodontic treatment and transplantation of premolars. Am J Orthod Dentofacial Orthop 2012;142:710–9. doi:10.1016/j.ajodo.2011.03.028.
